# Physiological Strategies to Improve the Performance of Spring Maize (*Zea mays* L.) Planted under Early and Optimum Sowing Conditions

**DOI:** 10.1371/journal.pone.0124441

**Published:** 2015-04-30

**Authors:** Muhammad Amir Bakhtavar, Irfan Afzal, Shahzad Maqsood Ahmed Basra, Azraf-ul-Haq Ahmad, Mehmood Ali Noor

**Affiliations:** 1 Department of Crop Physiology, University of Agriculture, Faisalabad-38040, Pakistan; 2 Department of Agronomy, University of Agriculture, Faisalabad-38040, Pakistan; 3 Department of Agricultural Extension and Rural Society, College of Food and Agriculture Sciences, King Saud University, Riyadh, Saudi Arabia; Henan Agricultural Univerisity, CHINA

## Abstract

Low temperature at stand establishment and high temperature at reproductive stage are involved in reduction of grain yield of spring maize. A field study was therefore conducted to evaluate different physiological strategies for improving performance of spring maize under temperature extremes. Seed priming and foliar spray with 3% moringa leaf extract (MLE) and 100 mg L^-1^ kinetin solution alone or in all possible combinations with each other at three growth stages (knee height, tasseling and grain filling stage) and hydropriming was compared with control. Seed priming plus foliar spray of MLE and kinetin significantly improved stand establishment especially under early sown crop as indicated by reduced mean emergence time (MET), improved emergence index (EI) and final emergence percentage (FEP). Similarly increased chlorophyll contents, crop growth rate, leaf area index, photosynthetic rate, transpiration rate, relative water content and decreased membrane permeability were recorded in both early and optimum sowing conditions in MLE priming plus foliar spray treatment. All these improvements were harvested in the form of increased yield and harvest index compared with control treatment. Overall crop sown at optimum time performed best but exogenous application of MLE through seed priming and foliar spray maximally improved the performance of early sown maize crop which is attributed more likely due to improved stand establishment, chlorophyll and phenolic contents, increased leaf area duration and grain filling period. It can be concluded that seed priming with MLE along with its foliar spray could increase production of maize under temperature extremes.

## Introduction

Out of 1.1 billion tons of coarse grains produced in the world during 2010 for food, feed and industrial purposes, maize accounts for 74% of aggregate output [1]. In spring planted maize, a high day temperatures of 38°C at reproductive stage directly affect pollination and seed setting resulting in reduced grain yield [2]. One possible option to avoid heat stress is early planting of spring maize. Furthermore early sowing results in bold and heavier grains due to increased grain filling duration [3]. Early planted spring maize has experienced a decrease in daily soil temperature below 10°C that causes imbibitional injury to germinating seed [4]. In temperate regions, low temperature stress at early developmental stages resulted in poor photosynthetic performance of maize [5], which further leads to retarded plant growth and development [6]. Under chilling stress, higher activities of reactive oxygen species has been observed that cause oxidative damage to membranes and impairs the normal functions of cells by reacting with proteins, lipids and DNA [7].

In spring planted maize damaging effects of low temperature at seeding stage and of high temperature on pollination and grain filling could be reduced by early plantation of short duration varieties that are tolerant to low or high temperature [8]. Seed enhancement techniques like seed priming resulted in improved germination and stand establishment under low temperature conditions [4]. Seed soaking in salicylic acid solution induced chilling tolerance in hybrid maize chiefly due to the activation of antioxidants [7]. Hydropriming lowered the time taken to 50% germination and higher germination index, vigor index and final germination percentage of maize genotypes [9]. Priming wheat seed with optimum concentration of kinetin solution reduced the devastating effects of salinity on wheat and resulted in higher seedling growth and increased fresh and dry weight under both saline and non-saline conditions [10]. Foliar application of growth promoting substances e.g. salicylic acid has been shown to increase photosynthetic rate [11], and maintain membrane integrity [12], in maize. Although plant growth and development is promoted by the proper exogenous application of plant hormones along with nutrients, antioxidants, organic and inorganic chemicals but these are expensive and out of reach of resource poor farmers. *Moringa oleifera* could be an alternative source of plant hormones as moringa leaves are rich source of antioxidants (phenolics, ascorbate), vitamins like (A, B, C), different essential minerals (K, Ca, Fe), proteins and zeatin [13]. Priming maize seed with moringa leaf extract reduces mean germination and increased germination index that ultimately improved seedling growth by increasing chlorophyll content, amylase activity and total sugar contents under chilling conditions [8]. Foliar spray of moringa leaf extract can extend seasonal leaf area duration, the grain-filling period and delay crop maturity that results into greater economic and biological yields of wheat crop under late sown conditions [14].

Until now studies have been carried out to investigate the individual effect of foliar spray and seed priming with kinetin and hydropriming in improving stand establishment and growth of maize under stress conditions especially salinity [15]. Present study was therefore carried out to explore the potential of exogenous application of moringa leaf extract and kinetin through seed priming and foliar spray for reducing the chilling stress at early stage and heat stress at late reproductive stage in spring maize under early and optimum sowing conditions.

## Materials and Methods

### Seed material and experiment details

Seed of spring maize hybrid 32-F10 was obtained from Pioneer Seed limited, Sahiwal, Pakistan. Initial seed moisture content was 12.4% on fresh weight basis. The study was conducted at Agronomic Research Area, University of Agriculture Faisalabad (31.41°N latitude and 73.08°E longitude, Faisalabad, Pakistan) during spring, 2014. Experiment was comprised of two sowing dates (22 January and 22 February) and nine physiological strategies (seed priming and foliar application). Priming and foliar techniques include hydropriming, seed priming with kinetin, seed priming with moringa leaf extract (MLE), water spray, foliar spray of kinetin, seed priming plus foliar spray of MLE and seed priming plus foliar spray of kinetin. Untreated seeds were taken as control treatment.

The experiment was laid out in Randomized Complete Block Design (RCBD) with split plot arrangement, randomizing the sowing dates in main plots while seed priming and foliar application techniques was randomized in sub-plots with three replicates keeping net plot size of 6.0 m × 3.0 m. There were total 96 plants in each experimental unit having four rows. Recommended seed rate of 30 kg ha^-1^ was used for sowing and fertilizer N: P: K (250:120:100 kg/ha) was applied. All other agronomic and plant protection measures were uniform for all treatments.

### Seed priming protocol

Seed priming treatments used in this study were selected from previous experiments [8], [16]. For hydropriming, seeds were soaked in distilled water, while for kinetin and MLE priming; seeds were soaked in 100 mg L^-1^ kinetin and 3% MLE solution for 24 hours with continuous aeration provided by an aquarium pump. After soaking, seeds re-dried closer to their original weight under shade. Fresh moringa leaves were collected from a mature moringa tree and juice was extracted by a locally fabricated juice extraction machine [13].

### Foliar spray

Fresh moringa leaf extract was diluted up to 3% and was applied with a hand sprayer at knee height, tasseling and grain filling stages to field-grown maize plot. Foliar spray of 100 mg L^-1^ kinetin solution was also applied using same procedure and equipment as described for MLE spray.

### Measurements

#### Emergence

Numbers of emerged seedlings were recorded daily according to the seedling evaluation handbook of the Association of Official Seed Analysis [17]. Mean emergence time (MET) was calculated according to the equation [18].

$$\text{MET }=\;\frac{\sum \text{Dn }}{\sum \text{n}}$$

Where n is the number of seedlings emerged on day D, and D is the number of days counted from the beginning of emergence. Final emergence percentage was calculated by counting final number of seedling emerged in all days and divided it by total number of seeds sown and then multiplied it by 100.

#### Growth and development

One square meter area containing four plants was harvested from each replicate at fortnight interval and fresh weight of harvested samples was recorded. 50 g sample comprised of stem, leaves and cobs in equal ratio was dried in oven for determination of dry weight. Crop growth rate (CGR) was measured using equation [19].

$$\text{CGR }=\;\frac{{\text{W}}_{2}-{\text{W}}_{1}}{{\text{t}}_{2}-{\text{t}}_{1}}$$

Where;

- CGR = Crop growth rate
- W_1_ = Total dry matter at the first harvest
- W_2_ = Total dry matter at the second harvest
- t_1_ = Date of observation of first dry matter
- t_2_ = Date of observation of second dry matter

Leaf area was measured using leaf area meter (Model: CI 203) and leaf area index was calculated using equation;

Leaf area index = Leaf area/Land area

#### Physiological attributes

Gas exchange measurements for photosynthic rate and transpiration rate were taken from ear leaf at silking stage. For this purpose a portable photosynthesis system (LCiAnalyzer with Broad Head, Part Number LCi-002/B and Serial Number 32455) was used. Three plants were tagged from each plot and measurements were taken on 6.25cm^2^ area in the central part of the leaf blade that did not include midrib. These measurements were made from 10.00 a.m. to 3.00 p.m in bright sunny day.

#### Biochemical analysis of ear leaf

Ear leaves were collected at silking stage for biochemical analysis as major contribution towards final yield is made by the ear leaf. Membrane stability was determined in terms of electrolyte leakage in leaf samples which were cut into six equal size segments [20]. Fresh leaves (0.5 g; W_f_) were rinsed in water until the weight of the leaves was constant. The saturated leaves were weighed (W_s_) and then dried for 24 h at 80°C for determinations of the dry weigh (W_d_). Relative water content (RWC) was calculated by using formula [21]:

$$\text{RWC }=\;\frac{{\text{W}}_{\text{f}}-{\text{W}}_{\text{d}}}{{\text{W}}_{\text{s}}-{\text{W}}_{\text{d}}}\times 100$$

For chlorophyll determination, leaf sample of 0.5 g was grinded in 5 mL of 80% acetone. Poured it in cuvettes and read at 663 and 645 OD’s using UV-spectrophotometer (UV-4000). Values were substituted in the following formula:

$$\begin{array}{l} \text{A }=\;\frac{\left[\left(0.0127\left(\text{OD }663\right)-0.00269\left(\text{OD}645\right)\right)*100\right]}{0.5}\;=\;\frac{\text{mg}}{\text{g}}\;\text{of fresh weight}\\ \text{B }=\;\frac{\left[\left(0.0229\left(\text{OD }645\right)-0.00468\left(\text{OD}663\right)\right)*100\right]}{0.5}\;=\;\frac{\text{mg}}{\text{g}}\;\text{of fresh weight} \end{array}$$

Total phenolic contents were determined at ear leaf stage [22]. 1 g plant material was taken and homogenized with 80% acetone solution and supernatant was taken after phase separation. 20 μl sample was taken in a test tube along with 1.58 ml distilled water, 100μl Folin-Ciocalteau (FC) reagent and 300 μl sodium carbonate solution. The test tubes were kept in water bath at 40°C for 30 minutes. After that the absorbance was measured at 765 nm by spectrophotometer (UV 4000).

#### Agronomic and yield attributes

Plants from two rows comprising an area of 6.0 m × 3.0 m were harvested at when grains became blackish at the point of attachment with cob, giving a sign of harvest maturity. Data of agronomic traits and yield components including number of grains per row, number of grains per cob, 1000-grain weight, grain yield, biological yield and harvest index were recorded following standard procedures. Plant samples were sun-dried for determination of biological yield and harvest index [19].

#### Grain quality analysis

Soxhlet fat extraction method was used for measurement of oil content in grain. Grain protein content was determined by using Micro Kjeldahl Method [23].

### Weather Data

**Fig 1:**
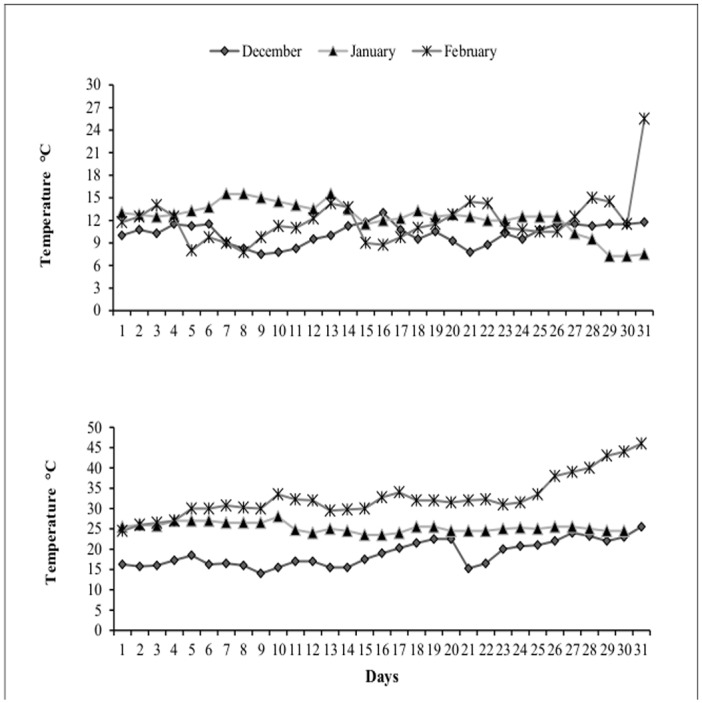
The daily fluctuation in average temperature during whole crop growth period of spring maize hybrid 32-F10.

The daily fluctuation in average temperature during whole crop growth period for early and optimal sowing of spring maize under climatic conditions of Faisalabad was as given in Fig 1.

### Economic analysis

Benefit to cost ratio for each treatment was calculated by dividing the gross income with total expenditures. Total expenditures for each treatment were calculated by the addition of total fixed cost (all the expenditures from sowing till harvesting) and total variable cost (different for each treatment including cost of exogenously applied agents). There was no cost of MLE and water spray except labor charges for exogenous application. The gross income was estimated using the prevailing average market prices of grain and stover in Pakistan.

### Statistical analysis

Data were analyzed statistically by using Fisher’s Analysis of Variance Technique and treatment means were compared by using Least Significantly Difference (LSD) test at 5% probability level. Emergence data were presented graphically in term of final emergence percentage using Microsoft Office Excel.

## Results

Suboptimal temperature decreased the emergence count in early sown maize crop by causing chilling injury to the growing seeds. Besides this realization that chilling stress considerably increased the mean emergence time and decreased final emergence percentage in early sown maize crop, seed priming with growth promoting substances like MLE and kinetin significantly reduced mean emergence time (MET) and increased emergence index (EI) and final emergence (FEP) under both early and optimum sowing conditions. Significant interaction was observed between treatments and sowing dates. Maximum final emergence percentage and emergence index while lower mean emergence time was observed in seeds primed with MLE and kinetin 100 mg L^-1^ under optimum sowing conditions (Table 1).

**Table 1:** Effect of seed priming and foliar application strategies on stand establishment of spring maize planted at early and optimum times.

Treatments	Mean emergence time (days)		Final emergence percentage		Emergence index	
	Early	Optimum	Early	Optimum	Early	Optimum
Control	14.48a	9.48d	72.22e	94.44ab	2.18n	2.31lm
Hydropriming	13.90b	9.13de	93.06ab	100.00a	2.87g	3.00f
Kinetin priming	14.17ab	9.12de	90.28bc	100.00a	3.40e	3.53d
MLE^1^ priming	14.13ab	9.18d	100.00a	100.00a	3.66c	3.79b
Water spray	14.29a	9.34de	79.32de	95.83ab	2.27mn	2.40kl
MLE^1^ spray	14.32a	9.25de	79.32de	95.37ab	2.53ij	2.66h
Kinetin spray	14.24ab	9.21de	75.46de	95.83ab	2.46jk	2.59hi
MLE^1^ priming plus foliar spray	13.23c	9.04e	100.00a	100.00a	3.84ab	3.94a
Kinetin priming plus foliar spray	13.91b	9.04e	100.00a	100.00a	3.83ab	3.83ab
LSD Interaction at P ≥ 0.05	0.36		8.65		0.12	

Means within a column followed by the same letters are not significantly different at P ≤ 0.05.

^1^MLE; Moringa leaf extract.

Likewise seed priming and foliar spray strategies significantly improved yield related traits and interaction between different strategies and sowing dates was found significant (Table 2). Among various growth promoting substances exogenous application of MLE through seed priming and foliar spray significantly increased cob diameter, number of grains per row and total number of grains per cob in both early and optimum sown crop. Maximum cob diameter, number of grains per row and total number of grains per cob were produced by early sown crop with MLE priming plus foliar application treatment followed by kinetin priming plus foliar application treatment while lowest values were recorded in control treatment.

**Table 2:** Effect of seed priming and foliar application strategies on agronomic traits of spring maize planted at early and optimum times.

Treatments	Cob diameter (cm)		Grains per row of cob		Grains per cob	
	Early	Optimum	Early	Optimum	Early	Optimum
Control	4.55i	4.59h	39.70c	34.60f	789.59j	802.84j
Hydropriming	4.63fg	4.65de	41.43a	36.64e	842.60hi	885.05e
Kinetin priming	4.63fg	4.64ef	41.67a	39.17cd	841.44hi	870.84ef
MLE^1^ priming	4.67cd	4.68c	41.87a	41.33ab	914.30cd	922.07c
Water spray	4.56i	4.58h	39.77c	38.27d	794.32j	804.57j
MLE^1^ spray	4.64e-g	4.68c	41.57a	39.10c	843.07hi	853.21gh
Kinetin spray	4.62g	4.63fg	40.23bc	40.20bc	830.27i	861.21fg
MLE^1^ priming plus foliar spray	4.75a	4.74a	42.33a	42.10a	978.53a	954.17b
Kinetin priming plus foliar spray	4.71b	4.72b	41.93a	41.80a	904.93d	930.30c
LSD Interaction at P ≥ 0.05	0.02		1.18		16.50	

Means within a column followed by the same letters are not significantly different at P ≤ 0.05.

^1^MLE; Moringa leaf extract.

Seed priming and foliar application resulted in significantly higher 1000-grain weight, grain and biological yields and harvest index as compared to control. Seed priming plus foliar spray of MLE showed best results both in early and optimum sown maize crop followed by seed priming and foliar application of kinetin (Table 3). As for economic analysis, MLE priming plus foliar spray was found cost effective as it gave maximum net income and cost to benefit ratio as compared to all other treatments (Table 4).

**Table 3:** Effect of seed priming and foliar application strategies on yield attributes of spring maize planted at early and optimum times.

Treatments	1000 grain weight (g)		Grain yield (t ha^-1^)		Biological yield (t ha^-1^)		Harvest index (%)	
	Early	Optimum	Early	Optimum	Early	Optimum	Early	Optimum
Control	259.77g	264.34f	4.43k	4.82i	11.83j	12.75 h	37.69h	37.72gh
Hydropriming	269.87e	272.13de	5.72e	5.74e	13.94c	13.89 d	41.00e	41.33de
Kinetin priming	272.97d	273.37d	5.64f	5.70ef	13.60f	13.66 e	41.50d	41.79d
MLE^1^ priming	279.03c	280.27c	6.10d	6.21c	14.57b	14.61 b	41.84d	42.50c
Water spray	261.69fg	264.13f	4.51j	4.84hi	11.84j	12.78 h	38.05gh	37.92gh
MLE^1^ spray	274.20d	279.93c	4.90h	5.70ef	12.81h	13.91 cd	38.21g	40.94e
Kinetin spray	272.03de	274.78d	4.87hi	5.44g	12.10i	13.46 g	40.29f	40.44f
MLE^1^ priming plus foliar spray	294.30a	293.10a	6.88a	6.87a	15.99a	15.98 a	43.06a	42.98ab
Kinetin priming plus foliar spray	289.23 b	290.00b	6.79b	6.86a	15.95a	15.96 a	42.57bc	42.97ab
LSD Interaction at P ≥ 0.05	3.04		0.07		0.05		0.55	

Means within a column followed by the same letters are not significantly different at P ≤ 0.05.

^1^MLE; Moringa leaf extract.

**Table 4:** Net income and benefit-to-cost ratio of spring maize hybrid 32F10 influenced by various physiological strategies.

Treatment	Total Expenditure (PKR^2^ ha^-1^)		Gross Income (PKR ha^-1^)		Net Income (PKR ha^-1^)		Benefit: Cost ratio	
	Early	Optimum	Early	Optimum	Early	Optimum	Early	Optimum
Control	93864	93864	112338	121954.5	18474	28090.5	1.20	1.30
Hydropriming	93864	93864	142047	142321.5	48183	48457.5	1.51	1.52
Kinetin priming	116364	116364	139734	141030	23370	24666	1.20	1.21
MLE^1^ priming	94464	94464	150817.5	153036	56353.5	58572	1.60	1.62
Water spray	94764	94764	114808.5	122409	20044.5	27645	1.21	1.29
MLE^1^ spray	95064	95064	123637.5	141592.5	28573.5	46528.5	1.30	1.49
Kinetin spray	202764	202764	121459.5	135549	-81304.5	-67215	0.60	0.67
MLE^1^ priming plus foliar spray	95364	95364	169105.5	168889.5	73741.5	73525.5	1.77	1.77
Kinetin priming plus foliar spray	225264	225264	167274	168651	-57990	-56613	0.74	0.75

^1^MLE; Moringa leaf extract.

^2^Pakistani Rupee = 0.01 United States Dollar

**Fig 2:**
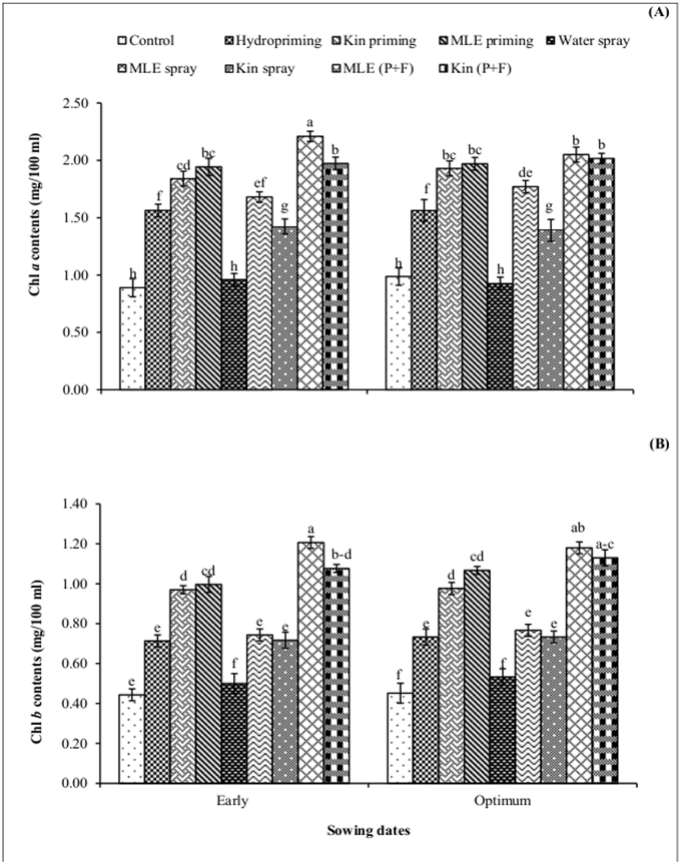
Chlorophyll content of spring maize in relation to exogenous application of growth promoting substances. (Kin; Kinetin, MLE; Moringa leaf extract, P+F; Priming followed by foliar spray). Vertical bars are standard error of means. Letters on the legends indicate significant (*P*<0.05) difference among physiological strategies.

**Fig 3:**
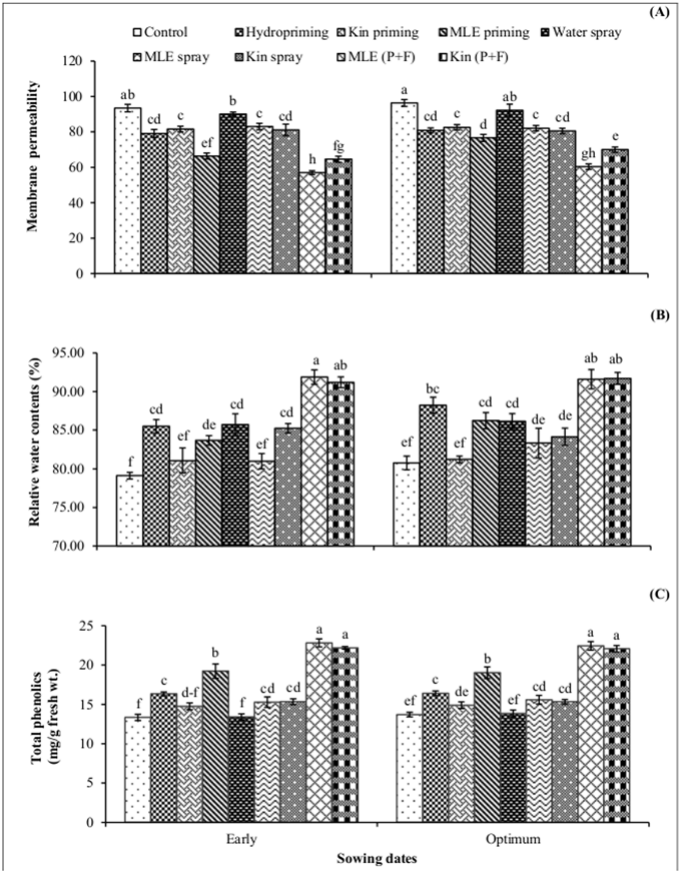
Membrane permeability, relative water content and total phenolics of spring maize in relation to exogenous application of growth promoting substances. (Kin003Binetin, MLE; Moringa leaf extract, P+F; Priming followed by foliar spray.) Vertical bars are standard error of means. Letters on the legends indicate significant (*P*<0.05) difference among physiological strategies.

All priming and foliar treatments increased chlorophyll a and b contents (Fig 2) in both early and optimum sowing conditions. However, maximum chlorophyll contents were obtained in MLE priming plus foliar treatment in early sown maize crop as compared to control treatment. Seed priming plus foliar spray of MLE in early sown maize crop significantly reduced the membrane permeability compared with control treatment in which higher values for membrane permeability was recorded (Fig 3A). High relative water content and total phenolics were obtained in MLE priming plus foliar spry treatment in maize crop sown in early conditions (Fig 3B and 3C).

**Fig 4:**
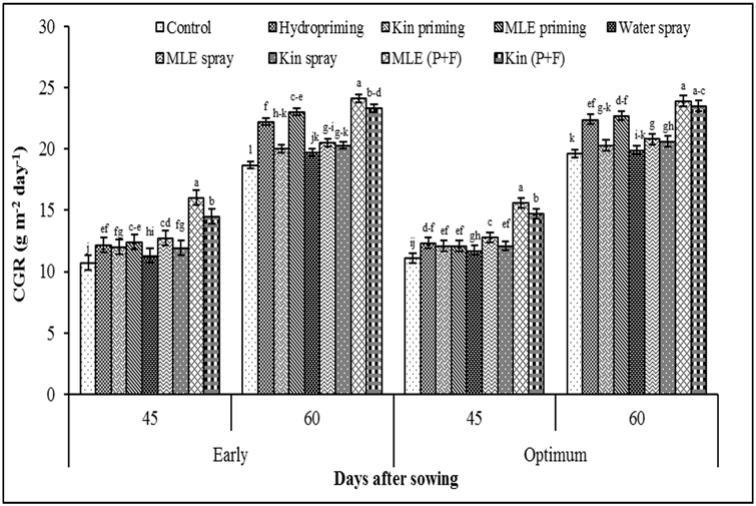
Crop growth rate of spring maize in relation to exogenous application of growth promoting substances. (Kin; Kinetin, MLE; Moringa leaf extract, P+F; Priming followed by foliar spray.) Vertical bars are standard error of means. Letters on the legends indicate significant (*P*<0.05) difference among physiological strategies.

**Fig 5:**
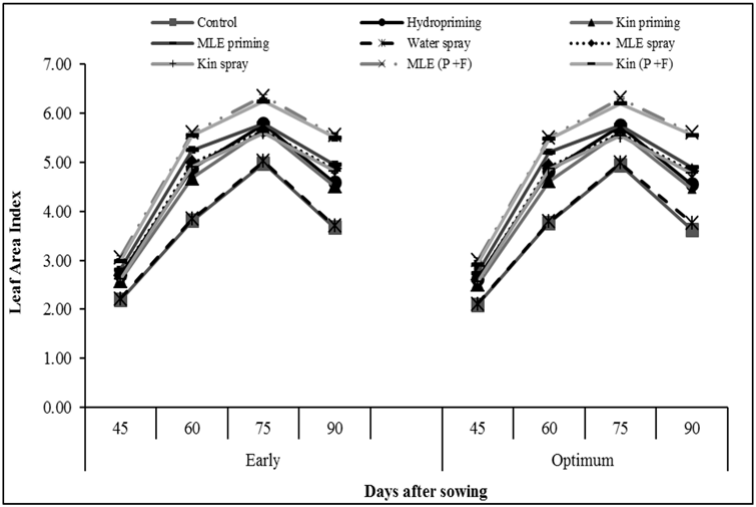
Leaf area index of spring maize in relation to exogenous application of growth promoting substances. (Kin; Kinetin, MLE; Moringa leaf extract, P+F; Priming followed by foliar spray.)

**Fig 6:**
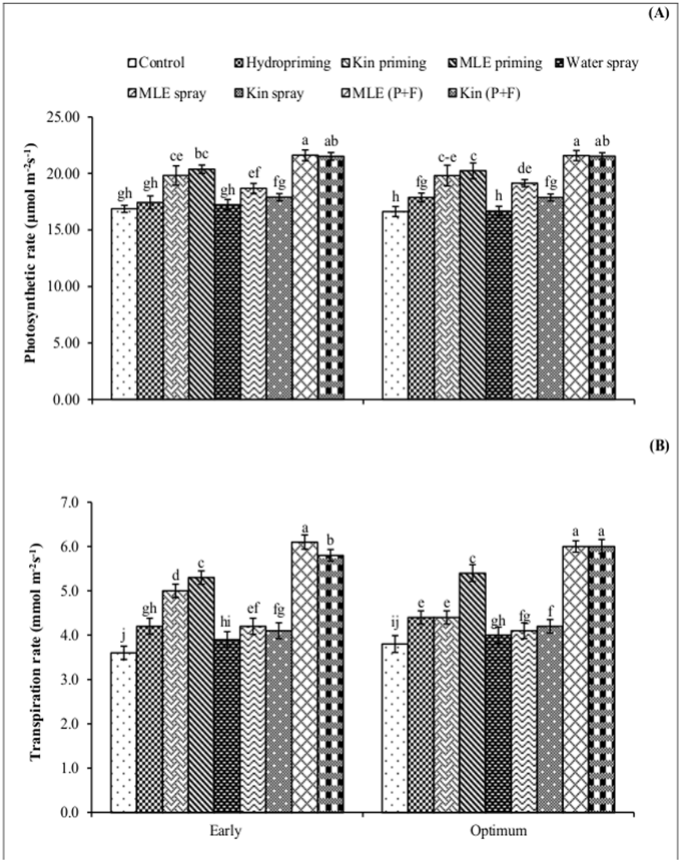
Photosynthetic rate and transpiration rate of spring maize in relation to exogenous application of growth promoting substances. (Kin; Kinetin, MLE; Moringa leaf extract, P+F; Priming followed by foliar spray.) Vertical bars are standard error of means. Letters on the legends indicate significant (*P*<0.05) difference among physiological strategies.

**Fig 7:**
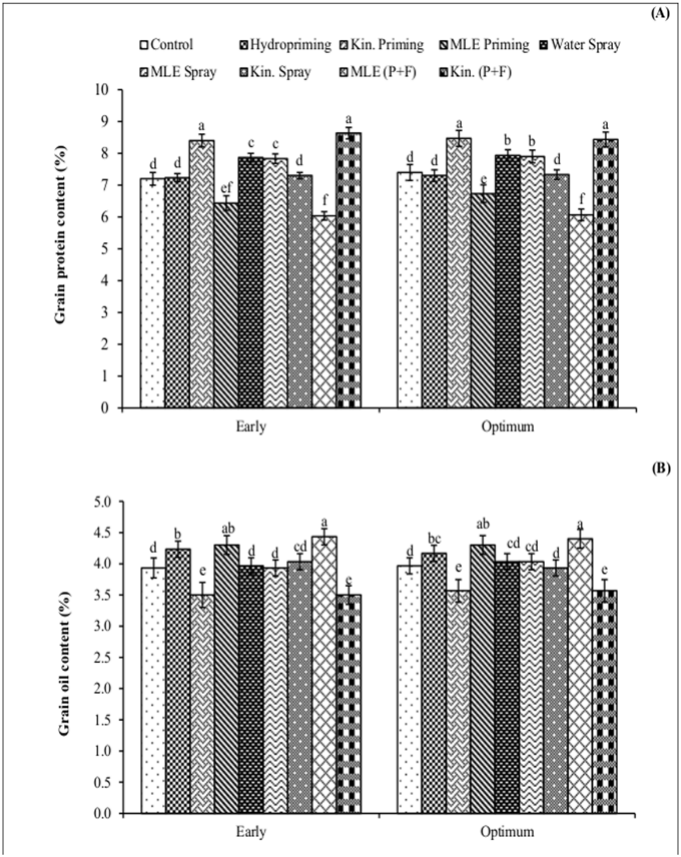
Grain quality attributes of spring maize in relation to exogenous application of growth promoting substances. (Kin; Kinetin, MLE; Moringa leaf extract, P+F; Priming followed by foliar spray.) Vertical bars are standard error of means. Letters on the legends indicate significant (*P*<0.05) difference among physiological strategies.

Crop growth rate recorded at 45 and 60 days after sowing was higher in response to application of MLE through seed priming and foliar spray (Fig 4). Likewise, higher values for leaf area index were also recorded for combined application of MLE through seed priming and foliar spray (Fig 5). Exogenous application of MLE through seed priming and afterward foliar spray in both early and optimum sowing condition increased the photosynthetic rate (Fig 6A) and transpiration rate (Fig 6B). Maximum grain protein contents were measured in kinetin priming alone and kinetin priming plus foliar spray treatments while higher values for oil contents were recorded from grains harvested from MLE priming plus foliar spray treatment under both early and optimum conditions (Fig 7).

## Discussion

Uniform emergence with higher final emergence count is the key foundation, which ensures the improvement of seedling performance and overall growth of crop. This study shows that seed priming treatments not only improved the stand establishment but also provided an energetic start to maize seedling under low temperature conditions (Table 1). A significant decrease in mean emergence time and increase in final emergence (Table 1) may be attributed to the fact that seed priming triggers a range of biochemical changes such as enzyme activation, starch hydrolysis and dormancy breaking in seed [24]. All these processes are required to start the germination process; due to this fact primed seeds have an edge over non-primed (control) which ultimately results in improved field emergence leading to better crop stand (Table 1) and increased crop growth rate (Fig 4).

Chilling stress in maize plants leads to the generation of ROS, which causes oxidative damage and impairing the optimal cellular functions by reacting with important macromolecules [7]. Seed priming along with its foliar spray increased total phenolics content in maize plant (Fig 3C). Phenolics have beneficial role in plant defense against biotic and biotic stresses like oxidative burst, improvement of vigor and germination under stress [25], [26]. Phenolics acts as hydrogen donators, reducing agents and quenchers of singlet oxygen, due to these facts phenolics may help in detoxification of reactive oxygen species [27]. Furthermore, improved relative water content and reduced membrane permeability in maize plants treated with MLE (seed priming plus foliar spray) might be due to maintenance of tissue water contents, increase in carbohydrate metabolism and antioxidant activities [7]. High crop growth rate, leaf area index (Figs 4 and 5) and higher photosynthetic rate (Fig 6A) by priming maize seed with MLE and kinetin was the result of earlier start of germination, better crop stand and vigorous growth (Table 1), confirming earlier findings that seed priming with MLE enhances maize performance under cool conditions [8]. Furthermore foliar spray of MLE also boost the growth rate of crop by increasing leaf area and photosynthetic rate as mineral composition of MLE makes it an excellent natural growth promoting substance influencing physiological processes going on within plant in a positive way [28]. Role of MLE in improving physiological attributes and yield of tomato has been reported [29], and similar results for improvement in leaf area of late sown wheat have been observed [14].

Seed priming resulted in rapid and uniform germination which leads to the production of vigorous seedlings with high chlorophyll contents in their leaves [30]. In present study exogenous application of MLE and kinetin through seed priming and foliar spray increased chlorophyll contents in maize plants (Fig 2) corresponds to earlier findings that seed priming with MLE delayed senescence under suboptimal conditions due to key role played by zeatin, ascorbate and minerals including potassium which are present in moringa leaves [16].

All the improvements in stand establishment, crop growth and phenology were harvested in the form of increased 1000-grain weight, better grain and biological yield, and higher values of harvest index (Table 3). Foliar spray of MLE applied at critical growth stages, especially knee height, tasseling and grain filling stages resulted in heavier and bold grain, increased grain and biological yield. These results are supported by study [14], that due to the presence of growth-promoting substances, foliar spray of moringa leaf extract can extend seasonal leaf area duration, the grain-filling period and delay crop maturity that ultimately resulted into greater economic and biological yields of wheat crop under late sown conditions. The nutrient profile, vitamin and mineral composition of different parts of moringa tree propose a key role of its aqueous extract in physiology and growth of maize as it contains vitamin A, vitamin B-Complex, C and nutrient such as phosphorus, potassium, magnesium, iron, chromium, copper, manganese and zinc [31].

Different nutrients, vitamins, minerals and growth promoting substances present in MLE boosted the crop growth rate and photosynthetic rate and ultimately more photo assimilates were translocated towards grain increasing its oil contents. The lower protein contents in the grains from MLE priming plus foliar spray treatment might be due to the fact that in developing seed more photosynthates were translocated towards lipid biosynthesis. Similar inverse relation for oil and protein contents has been reported [32].

## Conclusion

Of various physiological strategies, priming of maize seed with 3% MLE along with its foliar spray at knee height, tasseling and grain filling stage was most effective in improving stand establishment, growth, biological yield, grain yield and grain quality under both early and optimum sowing conditions. Furthermore among the various growth promoting substances used for priming and foliar spray MLE seems to be more practical being least expensive (Table 4), non toxic and most effective in improving growth performance and yield of maize. Seed priming plus foliar spray of kinetin was also statistically at par but its high cost made it practically impossible.

## References

[1] FAO. Trends in the Crop Sector (2010) Available: FAO. http://www.fao.org/docrep/015/i2490e/i2490e03b.pdf

[2] Wahid A, Perveen M, Gelani S, Basra SMA (2007) Pretreatment of seed with H_2_O_2_ improves salt tolerance of wheat seedlings by alleviation of oxidative damage and expression of stress proteins. J Plant Physiol 164:283–294. 16545492 10.1016/j.jplph.2006.01.005

[3] Rizzardi MA, Witech D, Deggerone I (1994) Grain yield and yield components of maize cultivars at two sowing dates. Ciencia Rural. 24: 477–482.

[4] Afzal I, Basra SMA, Shahid M, Saleem M (2008) Physiological enhancements of spring maize (*Zea mays* L.) under cool conditions. Seed Sci Technol. 36:497–503

[5] Stirling CM, Nie GY, Aguilera C, Nugawela A, Long SP, Baker NR (1991) Photosynthetic productivity of an immature maize crop: changes in quantum yield of CO_2_ assimilation, conversion efficiency and thylakoid proteins. Plant Cell Environ. 14: 947–954.

[6] Cohn MA, Obendorf RL (1978) Occurrence of a stelar lesion during imbibitional chilling of maize. Amer J Bot. 65: 50–56.

[7] Farooq M, Aziz T, Basra SMA, Cheema MA, Jabran K, BM Khan (2008) Chilling tolerance in hybrid maize induced by seed priming with salicylic acid. J Agron Crop Sci. 194: 161–169.

[8] Afzal I, Hussain B, Basra SMA, Rehman H (2012) Priming with moringa leaf extract reduces imbibitional chilling injury in spring maize. Seed Sci Technol. 40:271–276.

[9] Dezfuli PM, Janmohammadi M, Sharifzadeh F (2008) Influence of priming techniques on seed germination behavior of maize inbred lines (*Zea mays* L.). ARPN J Agric Biolo Sci. 3:22–25.

[10] Afzal I, Basra SMA, Iqbal A (2005) The effects of seed soaking with plant growth regulators on seedling vigor of wheat under salinity stress. J Stress Physiol Biochem. 1:6–14.

[11] Khan W, Prithiviraj B, Smith DL (2002) Photosynthetic responses of corn and soybean to foliar application of Salicylates. J Plant Physiol. 160:485–492. 10.1078/0176-1617-00865 12806776

[12] Kaya C, Tuna CL, Okant AM (2010) Effect of foliar applied kinetin and indole acetic acid on maize plants grown under saline conditions. Turk J Agric. 34: 529–538.

[13] Foidle N, Makkar HPS, Becker K (2001) The Potential of *Moringa oleifera* for agricultural and industrial uses In: Fuglie LJ, editor. The Miracle Tree: The Multiple Attribute of Moringa. pp. 45–76.

[14] Yasmeen A, Basra SMA, Ahmad R, Wahid A (2012) Performance of late sown wheat in response to foliar application of *Moringa oleifera* leaf extract. Chilian J Agric Res 72: 92–97.

[15] Ding D, Zhang L, Wang H, Liu Z, Zhang Z, Zheng Y (2009) Differential expression of miRNAs in response to salt stress in maize roots. Ann Bot. 103:29–38. doi: 10.1093/aob/mcn205 18952624 10.1093/aob/mcn205 PMC2707283

[16] Basra SMA, Iftikhar MN, Afzal I (2011) Potential of moringa (*Moringa oleifera*) leaf extract as priming agent for hybrid maize seeds. Int J Agri Biol. 13:1006–1010.

[17] AOSA (1983) Seed Vigor Testing Handbook Contribution No. 32 to the Handbook on Seed Testing. Association of Official Seed Analysts Springfield, IL.

[18] Ellis RA, Roberts EH (1981) The quantification of ageing and survival in orthodox seeds. Seed Sci Technol. 9:373–409.

[19] Hunt R (1978) Plant growth analysis Studies in biology No. 96. Edward Arnlod London, UK pp. 8–38.

[20] Blum A, Ebercon A (1962) Cell membrane stability as a measure of drought and heat tolerance in wheat. Crop Sci. 1981; 21: 43–47.

[21] Barr HD, Weatherley PE (1962) A re-examination of the relative turgidity technique for estimating water deficit in leaves. Aust J Biol Sci. 15, 413–428.

[22] Waterhouse AL (2003) Determination of total phenolics. Curr Protoc Food Anal Chem 11.1.1–11.1.8. doi: 10.1002/0471142913.faa0101s06

[23] AOAC (1990) Official methods of analysis, 15th ed Association of official analytical chemist. Virginia Inc; USA pp. 770–771.

[24] Aziza A, Haben A, Becker M (2004) Seed priming enhances germination and seedling growth of barley under condition of P and Zn deficiency. J Plant Nutr Soil Sci. 167:630–636.

[25] Parida A, Das AB, Sanada Y, Mohanty P (2004) Effects of salinity on biochemical components of the mangrove *Aegiceras corniculatum* . Aquatic Bot. 80: 77–87.

[26] Randhir R, Lin YT, Shetty K (2004) Phenolic, their antioxidant and antimicrobial activity in dark germinated fenugreek sprouts in response to peptide and phytochemical elicitors. Asia Pacific J Clin Nutr. 13: 295–30. 15331344

[27] Rice-Evans C, Miller NJ, Paganga G (1997) Antioxidant properties of phenolic compounds. Trends Plant Sci. 2:152–159.

[28] Anjorin TS, Ikokoh P, Okolo S (2010) Mineral composition of *Moringa oleifera* leaves, pods and seeds from two regions in Abuja, Nigeria. Int J Agric Biol.12: 431–434.

[29] Muhamman MA, Auwalu BM, Manga AA, Jibrin JM (2013) Effects of Aqueous extract of Moringa (*Moringa oleifera* Lam.) and Nitrogen rates on some Physiological attributes and yield of Tomato. Int J Chem Environ Biolo Sci. 1:2320–4087.

[30] Ghassemi-Golezani K, Khomari S, Valizadeh M, Alyari H (2008) Changes in chlorophyll content and fluorescence of leaves of winter rapeseed affected by seedling vigor and cold acclimation duration. J Food Agric Environ. 6:196–199.

[31] Fuglie LJ (2001) The Miracle Tree: *Moringa oleifera*: Natural Nutrition for the Tropics. Church World Service, Dakar pp.68.

[32] Truong Q, Koch K, Yoon JM, Everard JD, Shanks JV (2013) Influence of carbon to nitrogen ratios on soybean somatic embryo (cv. Jack) growth and composition. J Exp Bot. 1:1–13. 10.1093/jxb/ert138 PMC3697947 23740932

